# Supplementary material for: Quantum phase synchronization via exciton-vibrational energy dissipation sustains long-lived coherence in photosynthetic antennas
Source: Nat Commun. 2024 Apr 12;15:3171. doi: 10.1038/s41467-024-47560-6 (PMC11015008; doi:10.1038/s41467-024-47560-6)
Supplement: Supplementary file 1 — Supplementary Information [file 41467_2024_47560_MOESM1_ESM.pdf]

Supplementary Information

Quantum Phase Synchronization via Exciton-Vibrational Energy Dissipation Sustains Long-lived Coherence in Photosynthetic Antennas

Ruidan Zhu[1†], Wenjun Li[2†], Zhanghe Zhen[1,2†], Jiading Zou[1,3], Guohong Liao[1,3], Jiayu Wang[1,3], Zhuan Wang[1], Hailong Chen[1,3,4], Song Qin[2,3*], Yuxiang Weng[1,3,4*]

[1]Laboratory of Soft Matter Physics, Institute of Physics, Chinese Academy of Sciences, Beijing 100190, P.R. China;

[2]Yantai Institute of Coast Zone Research, Chinese Academy of Sciences, Yantai 264003, P. R. China;

[3]University of Chinese Academy of Sciences, Beijing 100049, P.R. China;

[4]Songshan Lake Materials Laboratory, Dongguan 523808, Guangdong, P.R. China.

[†]These authors contributed equally to this work.

*Corresponding authors:

Yuxiang Weng (Email: yxweng@iphy.ac.cn); Song Qin (Email: sqin@yic.ac.cn)

Table of Contents

Supplementary Note 1: Sample preparation and pulse characterization 2

Supplementary Note 2: Supplementary circular dichroism and Raman spectra 5

Supplementary Note 3: Supplementary 2D spectra of the monomer and β subunit........7

Supplementary Note 4: Determination of diagonal and cross peaks in 2D spectra of the trimer 7

Supplementary Note 5: Global analysis of the spectral evolution in 2DES 8

Supplementary Note 6: Kinetic analysis of the excited-state absorption signal............10

Supplementary Note 7: Supplementary analysis of dynamical Stokes shift data..........11

Supplementary Note 8: Supplementary HDTG and BBTA measurement data.............13

Supplementary Note 9: Supplementary assignments of vibrational modes...................16

Supplementary Note 10: Rephasing coherence maps for the rAPC trimer and α subunit..17

Supplementary Note 11: Detailed descriptions of the theoretical model 18

Supplementary References...24

Supplementary Note 1: Sample preparation

The two plasmids (p1 and p2) for expression of the recombinant allophycocyanin (rAPC) trimer were constructed as described previously[1]. Briefly, the double plasmids were co-transfected into *Escherichia coli* cells, allowing for intracellular expression and self-assembly of the trimeric protein (Supplementary Fig. 1a). The double plasmids were then modified to enable heterologous recombinant expression of the apcA (α-subunit) (Supplementary Fig. 1b) and apcB (β-subunit) (Supplementary Fig. 1c) in *E. coli*.

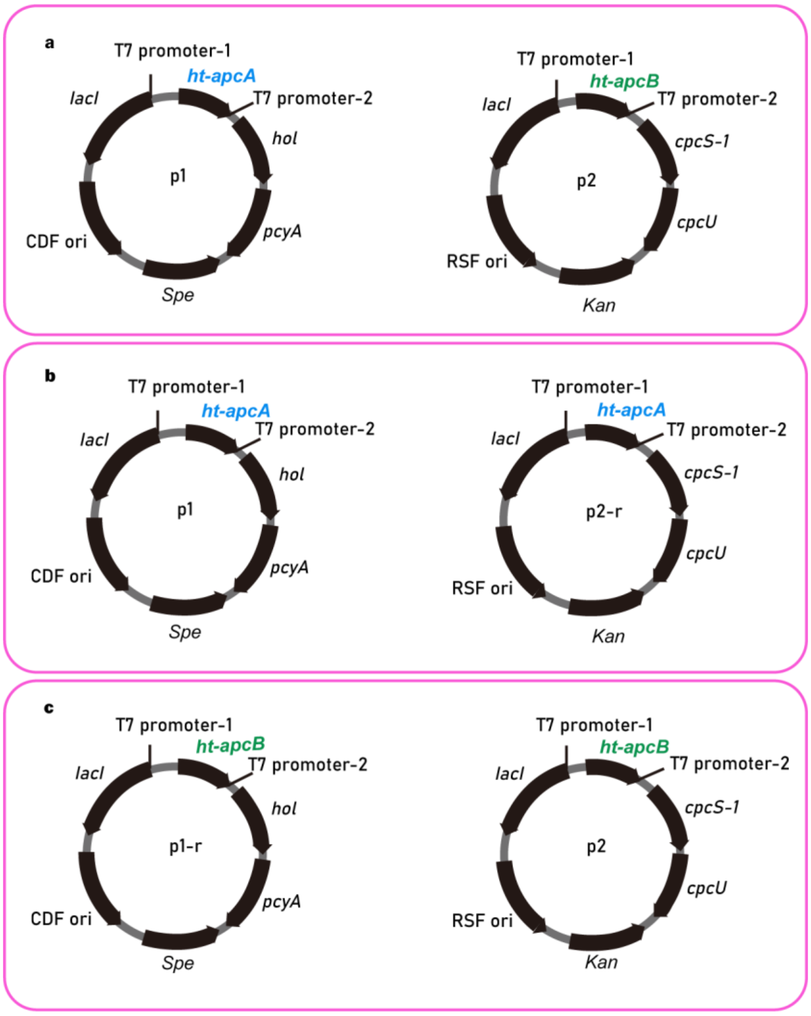

Supplementary Figure 1. Plasmid maps. (a) Map of plasmid (p1 and p2) used for inducible expression of the rAPC trimer. **(b)** Map of plasmid (p1 and p2-r) used for inducible expression of the of the α subunit. **(c)** Map of plasmid (p1-r and p2) used for inducible expression of the of the β-subunit. p1-r: The *ht-apcA* in p1 was replaced by *ht-apcB*; p2-r: The *ht-apcB* in p2 was replaced by *ht-apcA*.

The rAPC trimer, α-subunit, and β-subunit were prepared as described in our previous studies[1], with slight modifications. The *E. coli* cells were cultured at 37°C, and the final concentration of each antibiotic was 50 μg/mL. Recombinant protein expression was induced by 4 mM α-Lactose monohydrate at 28 °C for 18 h in cultures with an optical density between 0.6–0.7 at 600 nm. *E. coli* cells were harvested by centrifugation at 8000g for 10 min, washed and

suspended in 20 mM sodium phosphate (pH 7.4). The cells were broken up at 1000 bar by a high-pressure cell crusher (JNBIO JN-2.5, China). Recombinant proteins were purified using a Ni sepharose HP column (Cytiva, US). Purification of the trimer was followed by discontinuous sucrose density gradient centrifugation (Sucrose densities: 0.25 M, 0.5 M, 0.75 M, 1.0 M, 1.5 M. Sucrose was dissolved in 750 mM phosphate (pH 7.4) (Supplementary Fig. 2a). The rAPC monomer was prepared by disassembling the rAPC trimer using 300 mM imidazole with 20 mM phosphate and further purified by sucrose density gradient centrifugation (Supplementary Fig. 2b). (Sucrose densities: 0.25 M, 0.5 M, 0.75 M, 1.0 M, 1.5 M. Sucrose was dissolved in 20 mM phosphate (pH 7.4).)

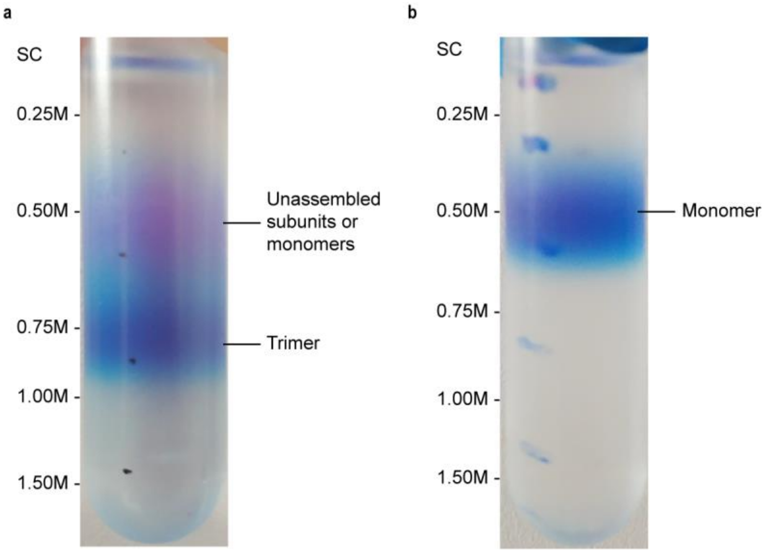

Supplementary Figure 2. Discontinuous sucrose density gradient separation of rAPC trimers and monomers. (**a**) The isolated rAPC trimer is in the 0.75 M sucrose layer. (**b**) The isolated rAPC monomer is in the 0.5 M sucrose layer. SC: sucrose concentration.

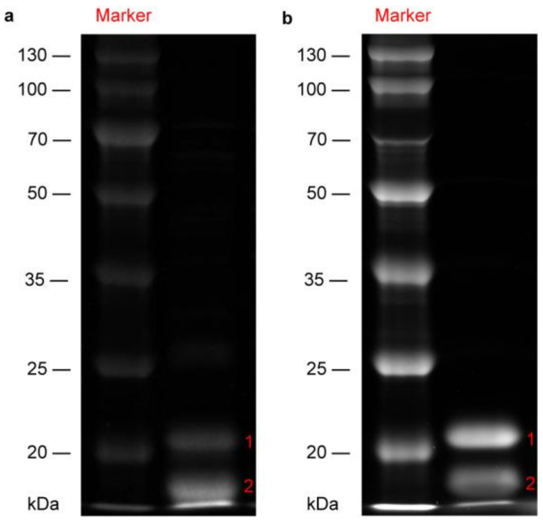

Supplementary Figure 3. SDS-PAGE identification of the rAPC trimer. (**a**) SDS-PAGE gel visualized by Coomassie blue staining. (**b**) SDS-PAGE gel visualized under DyLight650 illumination to verify the fluorescence of rAPC. 1: α-subunit with a His tag, 21 kDa; 2: β-subunit

with a His tag, 19 kDa; Marker: molecular mass standard.

Sodium dodecyl sulfate-polyacrylamide gel electrophoresis (SDS-PAGE) was used for protein identification (Supplementary Figs. 3 and 4). The results of SDS-PAGE were checked by Bio-Rad Chemidoc Imaging System (Bio-Rad, US), and the SDS-PAGE results are consistent with previous reports[2]. The linear absorption spectra of rAPC samples are shown in Supplementary Fig. 5, recorded by a commercial absorption spectrometer (Avantes). The rAPC monomer and two subunits have almost identical and broad spectral line shapes with absorption maxima around 618 nm. rAPC trimers show a red-shifted absorption maximum at 650 nm and a pronounced shoulder at about 624 nm. These results are in general agreement with the spectral features of natural APC.

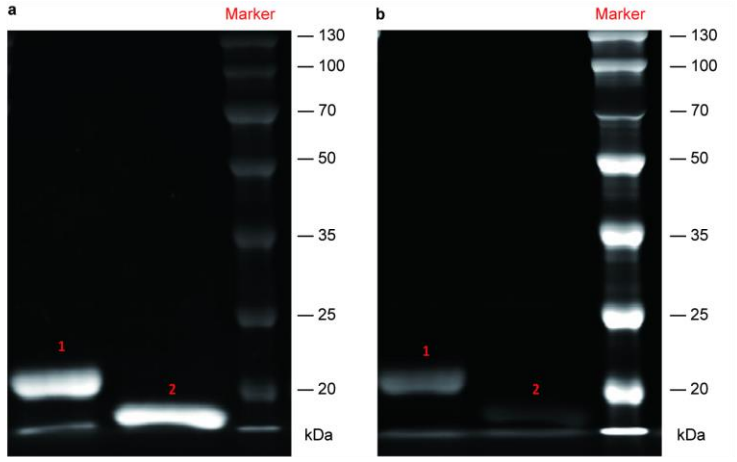

Supplementary Figure 4. SDS-PAGE identification of rAPC subunits. (a) SDS-PAGE gel visualized by Coomassie blue staining. **(b)** SDS-PAGE gel visualized under DyLight650 illumination to verify the fluorescence of samples. 1: α-subunit with a His tag, 21 kDa; 2: β-subunit with a His tag, 19 kDa; Marker: molecular mass standard.

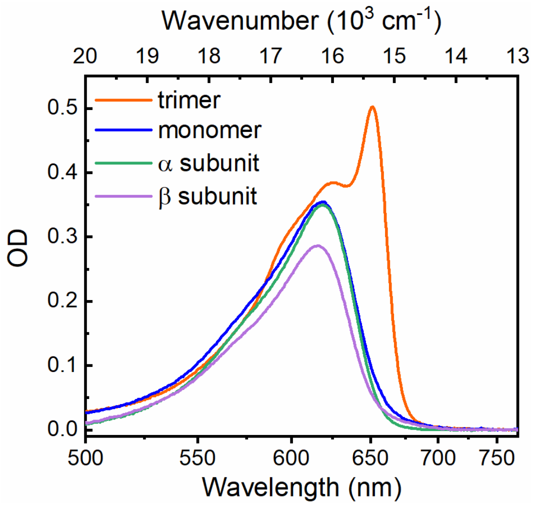

Supplementary Figure 5. Linear absorption spectra of the rAPC trimer, monomer, α-and β-subunits. These samples are contained in 1-mm path length cuvettes at room temperature. The rAPC monomer and two subunits have the same absorption maximum around 618 nm, while trimers show a red-shifted absorption maximum at 650 nm. Source data are provided as a Source

Data file.

A home-built non-collinear optical parametric amplifier (NOPA) pumped by a Ti:sapphire laser (Spitfire Ace; Spectra-Physics) with a 1-kHz repetition rate generated the broadband laser pulses spanning from 550 to 720 nm. The laser pulses were compressed by the combination of a grating pair and a fused silica prism pair. The pulse duration determined at the sample position by transient-grating frequency-resolved optical gating (TG-FROG) was ~8 fs (Supplementary Fig. 6).

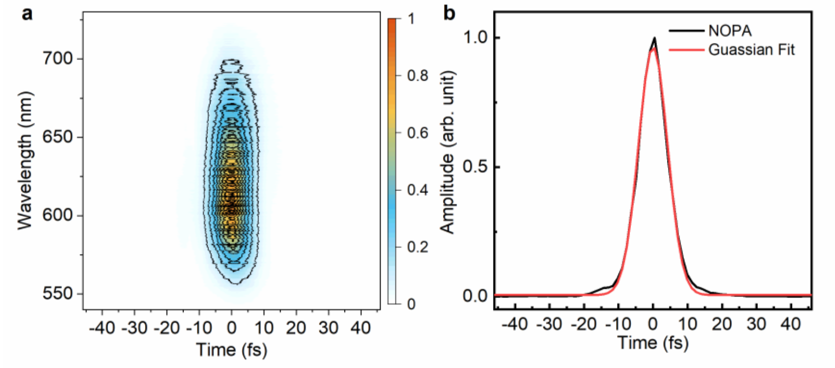

Supplementary Figure 6. Laser pulse characterization. (**a**) TG-FROG measurements were performed for a buffer solvent. The TG-FROG map was recorded under the same experimental conditions as used for 2DES. Contour lines are drawn in 10% intervals. (**b**) An integrated TG-FROG trace along the wavelength axis (black) was fitted by a Gaussian function (red), giving rise to a pulse duration of 8.1 fs (considering a deconvolution factor of 1.22 for TG-FROG). Source data are provided as a Source Data file.

Supplementary Note 2: Supplementary circular dichroism and Raman spectra

2.1 Determination of the electronic coupling strength between neighboring α-and β-pigments in the rAPC trimer

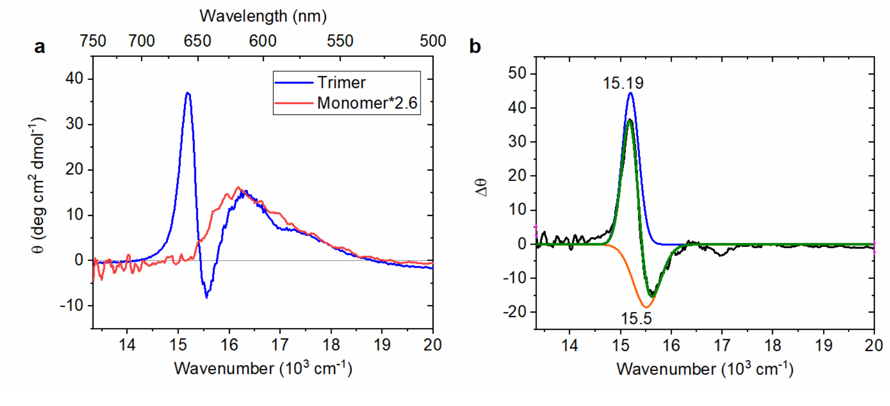

Supplementary Figure 7. Circular dichroism spectra of the rAPC trimer and monomer. (**a**) CD spectra of the rAPC trimer (blue) and monomer (red) in the visible range. (**b**) The difference CD spectrum between the trimer and monomer revealing the excitonic contribution, shown as a black line, is fitted well by the sum (green) of two Gaussian peaks with positive (blue) and negative (orange) amplitudes. Source data are provided as a Source Data file.

Circular dichroism (CD) spectra of the trimer and monomer (Supplementary Fig. 7a) were measured at room temperature on a Chirascan-plus circular dichroism spectrometer (Applied Photophysics, U.K.) in a 1 mm pathlength quartz cuvette. The CD spectrum (red) of the monomer, which is without excitonic interaction, was subtracted from that (blue) of the trimer with a scaling factor of 2.6. The resulting lineshape in Supplementary Fig. 7b was fitted with two Gaussian peaks to determine the excitonic splitting. This excitonic splitting energy obtained (310 cm^{-1}) is similar to that obtained in previous work[3]. Since the excitonic CD band splitting is about twice the electronic coupling strength (J), we determined that $J \approx 155$ cm^{-1}.

2.2 Comparison of non-resonance Raman spectra for the rAPC trimer and monomer

The Raman spectra were recorded by a Labram HR evolution Raman spectrometer (Horiba, USA) at room temperature. All the samples were freeze-dried into powder to obtain a strong enough Raman signal from chromophores. The excited wavelength was 785 nm, which led to non-resonance excitation. Raman spectra of the trimer and monomer obtained after the removal of the background signal and smoothing the data via adjacent data point averaging are shown in Supplementary Fig. 8. For better comparison of Raman intensities, both spectra were normalized to the maximum intensity, which was observed at 1640 cm^{-1}.

In contrast to the significantly reduced coherent amplitude observed for all three vibrational modes (270, 660, and 805 cm^{-1}) in the integrated coherence spectra of trimers, only the low-frequency mode, 270 cm^{-1} showed a remarkable decrease in the Raman spectrum; the relative intensities of the vibrational frequencies 660 and 805 cm^{-1} (805 cm^{-1} was slightly blue-shifted to 820 cm^{-1} in the powder) in the near-resonant region were nearly unchanged. The difference in the Raman intensity reveals that the decrease in coherent amplitude at 270 cm^{-1} is primarily due to the reduced Huang-Rhys factor of excitons resulting from the conformational change of the α84 pigments after trimerization.

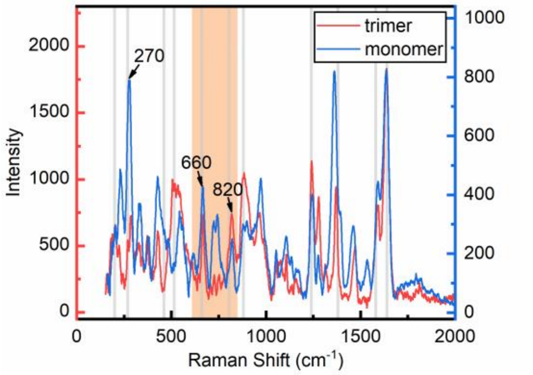

Supplementary Figure 8. Raman spectra of the rAPC trimer and monomer. Gray lines indicate vibrational modes observed in both spectra: 200, 270, 465, 515, 660, 805, 880, 1240, 1380, 1580, and 1640 cm^{-1}. The orange shaded area shows the near-resonant frequency region in the trimer. Source data are provided as a Source Data file.

Supplementary Note 3: Supplementary 2D spectra of the monomer and β-subunit

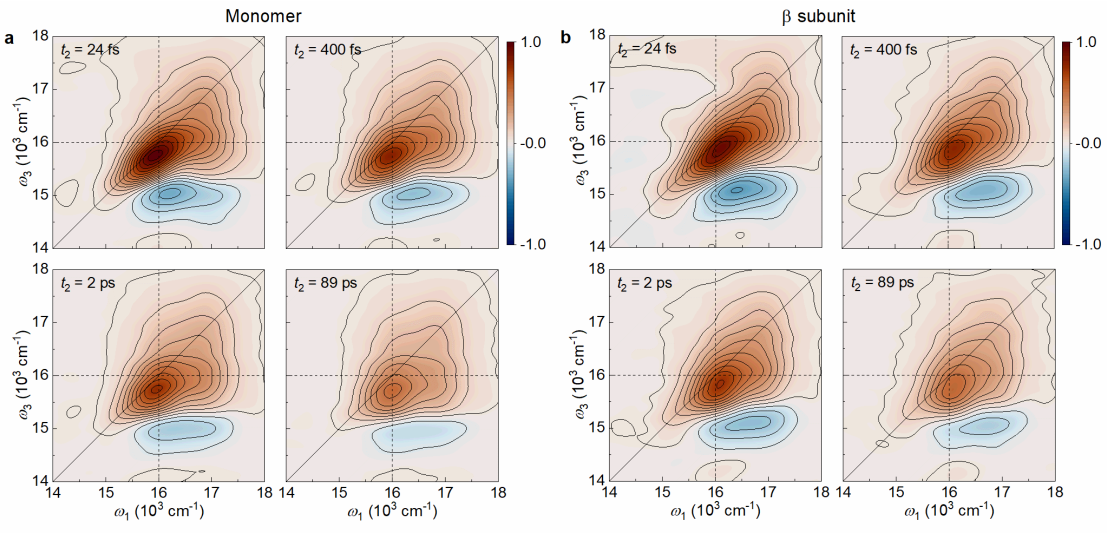

Supplementary Figure 9. Absorptive 2D spectra of the rAPC monomer (a) and β subunit (b). Dashed lines indicate the electronic transition energy. Contour lines are drawn in 7.8% intervals. Source data are provided as a Source Data file.

Supplementary Note 4: Determination of diagonal and cross peaks in 2D spectra of the trimer

In Supplementary Fig. 10, we plot the spectral slices along the diagonal on the 2D spectra of the trimer (shown in Fig. 2a in the main text) at different waiting times. In addition to the maximum diagonal peak of the lower exciton state at 15300 cm^{-1}, the diagonal peak of the upper exciton state at 16000 cm^{-1} is more clearly observed as shown by the arrow in Supplementary Fig. 10. The higher-energy peak near 17000 cm^{-1} comes from the vibronic transition of both exciton states. The upper diagonal peak at 16000 cm^{-1} decays more rapidly due to the energy transfer process so that it is barely visible in the spectral slices after 400 fs.

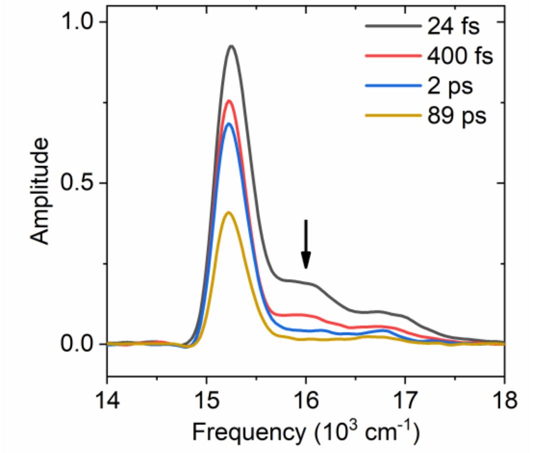

Supplementary Figure 10. The spectral slices along the diagonal on 2D spectra of the trimer at different waiting times. The arrow indicates the diagonal peak location of the upper exciton state. Source data are provided as a Source Data file.

The low distinction between these two diagonal peaks is due to the large inhomogeneous broadenings at room temperature, as shown in the linear absorption spectrum of the rAPC trimer (Fig. 1b in the main text). Therefore, we performed additional 2DES experiments at 77K to reduce the effect of inhomogeneous broadening. The rAPC trimer was dissolved in a 2:1 glycerol: buffer solution, and placed in a 500-μm quartz cell. An optical density of 0.45 was measured at 650 nm at 77 K. As shown in Supplementary Fig. 11, two diagonal peaks and the cross peak below the diagonal which corresponds to the energy transfer process can be clearly observed at low temperatures. The absorption peaks above the diagonal are also observed which can be assigned to the transitions of the excitonic levels to the higher excited states.

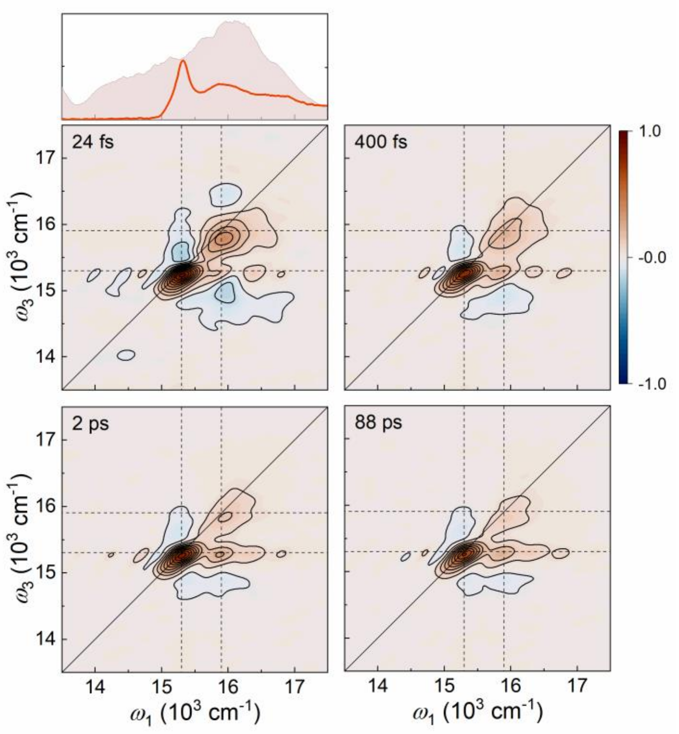

Supplementary Figure 11. Absorptive 2D spectra of the rAPC trimer at 77 K. Dashed lines indicate the electronic transition energy (15300 and 15900 cm^{-1}). Contour lines are drawn in 8.8 % intervals. The linear absorption spectrum (red) is shown on top of the 2D spectrum at 24 fs along with the laser spectrum (shaded area) used in the experiments. Source data are provided as a Source Data file.

Supplementary Note 5: Global analysis of the spectral evolution in 2DES

In the rAPC trimer, the spectral evolution over t_2 at two typical excitation frequencies, 15300 and 16000 cm^{-1}, is shown in detail in Supplementary Fig. 12a and 12b. Upon excitation at 15300 cm^{-1}, which corresponds to the peak absorption of the lower exciton in the trimer, the time-dependent spectra are similar. In addition to the overall decay of the spectra, the maximal peak undergoes a slight red shift from 15220 cm^{-1} to 15180 cm^{-1} and a slight spectral broadening. Global fitting from 40 fs to 95 ps revealed four lifetimes (130 fs, 1.6 ps, 57 ps, and 1.6 ns) in the

spectral evolution. The last lifetime, 1.6 ns, which corresponds to the reported ground-state population recovery lifetime due to fluorescence and radiationless decay, is a fixed constant during global analysis[4]. The second and the third lifetime constants may reflect the energy relaxation to the electronic ground state because their DAS exhibit a spectral profile similar to that of the longest component. The 130-fs component mainly corresponds to solvation processes, including dynamical Stokes shift and spectral diffusion. These two processes often co-occur so that the spectral features are mixed intensively and cannot be distinguished from each other. In addition to the exponential decay of the excited-state population, the kinetic trace of the diagonal peak at (15300, 15300) cm^{-1} (right panel of Supplementary Fig. 12a) also exhibits a large-amplitude coherent (oscillation) signal within 1 ps.

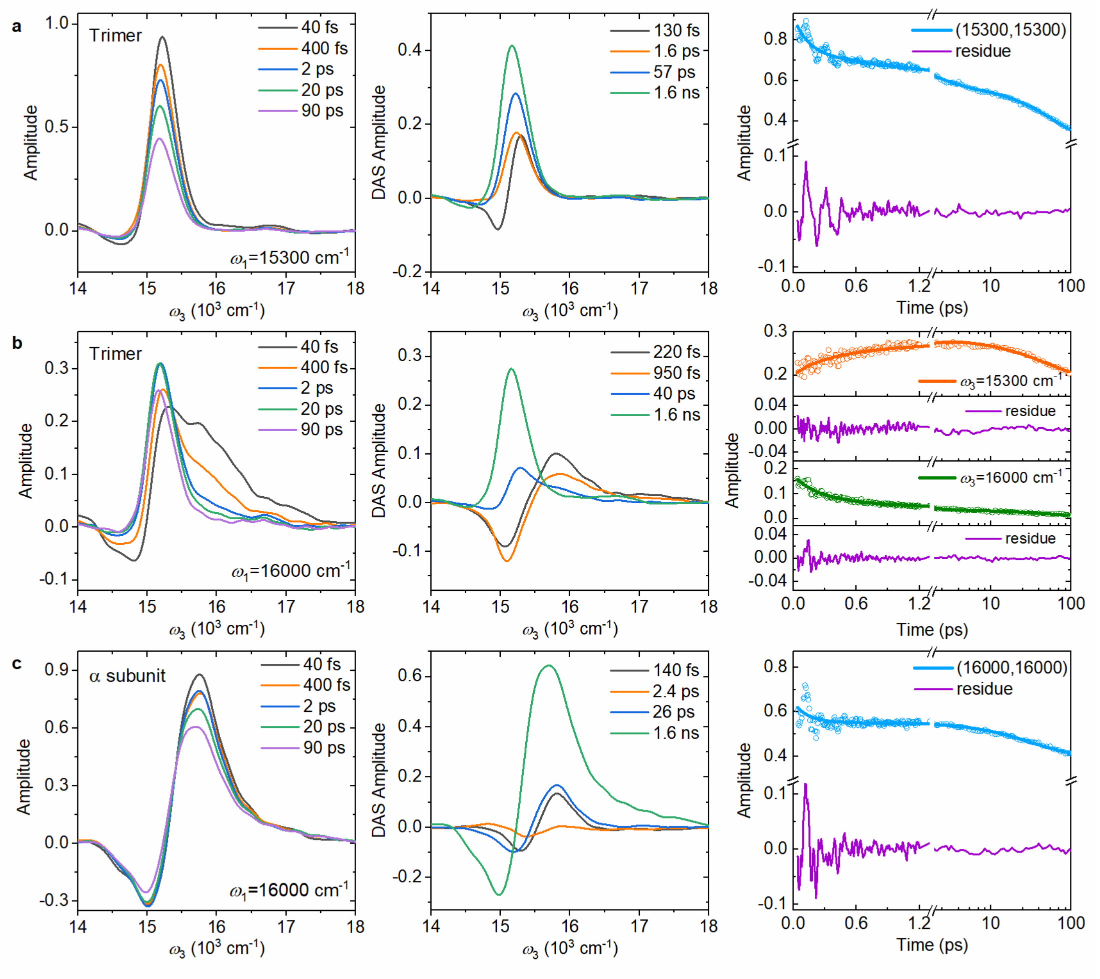

Supplementary Figure 12. Global analysis of spectral evolution at typical excitation frequencies for the trimer and the α-subunit. Spectral evolution and lifetime analysis at excitation frequencies of 15300 (**a**) and 16000 cm^{-1} (**b**) for the rAPC trimer and an excitation frequency of 16000 cm^{-1} (**c**) for the α-subunit. The spectra obtained at different waiting times are plotted in the left panels. Decay-associated spectra (DAS) with the corresponding lifetimes were obtained from a four-exponential fit of the sliced spectra from 40 fs to 95 ps in the middle panels. In the right panels, the open circles represent the measured data points, and the fitted lines on the symbols were obtained by global fitting. The residual dynamics (purple lines) indicate the

prominent oscillations within the first 1 ps. Source data are provided as a Source Data file.

Upon excitation at 16000 cm^{-1}, which corresponds to the upper exciton in trimer, the broad shoulder band decayed rapidly, within 400 fs, and nearly vanished after 2 ps. At longer waiting times, the amplitude of the main bleaching band decreased, and its spectral maximum shifted to the red slightly. Global fitting also revealed four lifetime constants (220 fs, 950 fs, 40 ps, and 1.6 ns) in the spectral evolution. DAS of the first two lifetime constants revealed the transfer of excitation energy from the upper exciton to the lower exciton in the trimer. The bi-exponential energy transfer process may arise from non-Gaussian microscopic heterogeneity in the distribution of energy transfer timescales, which was recently observed by the Schlau-Cohen group using single-molecule pump-probe spectroscopy[5]. Nonetheless, the first lifetime (220 fs) is closer to the timescale of energy transfer according to previous studies of the APC trimer.[6,7]

In the α-subunit, the spectral evolution over t_2 at the excitation frequency 16000 cm^{-1} is shown in detail in Supplementary Fig. 12c. The spectral evolution of the α-subunit is analogous to that of the trimer excited at 15300 cm^{-1} (Supplementary Fig. 12a), and DAS of the first two lifetime constants (140 fs and 2.4 ps) show a spectral lineshape similar to that of the fastest lifetime (130 fs) in the trimer corresponding to both Stokes shift and spectral diffusion processes. The third lifetime (26 ps) can be interpreted similarly to the third lifetime (57 ps) in Supplementary Fig. 12a as the energy relaxation to the ground state.

Supplementary Note 6: Kinetic analysis of the excited-state absorption signals

On the 2D spectra of rAPC, the peaks below the diagonal with negative amplitude are the excited-state absorption (ESA) peaks. For the monomer and two subunits, its central peak position locates at ω_1=16400, ω_3=14960 cm^{-1}; while for the trimer, two peaks locate at ω_1=16000, ω_3=14760 cm^{-1} (ESA-1) from the upper excitonic state and ω_1=15400, ω_3=14600 cm^{-1} (ESA-2) from the lower excitonic state, respectively. One of the major differences in the 2D spectra of the trimer and the monomer is the absence of the ESA-1 signal after 400 fs. The absence results from the ultrafast energy transfer process between the upper and lower excitonic state. The kinetics of these ESA signal are shown in Supplementary Fig. 13a. Through a three-exponential function fit from 40 fs to 95 ps, we extracted the fastest lifetime constants for these three traces: 142 ± 61 fs (monomer), 187 ± 115 fs (ESA-1), and 111 ± 59 fs (ESA-2). The fast decay of ESA-1 is consistent with the energy transfer lifetime of 220 fs, while the faster relaxation of monomer and ESA-2 corresponds to solvation process as elucidated in Supplementary Fig. 12.

To consider the compensation effect with the negative ESA signal for the data analysis of the monomer and two subunits, we separated its contributions into two parts: 1) for the decay kinetics, the ESA and the ground-state bleaching (GB)/excited-state emission (SE) peaks are well separated; 2) for the dynamical Stokes shift, the ESA (negative) and SE (positive) peaks on 2D spectra are all red-shifted as shown in Supplementary Fig. 13b. The results in Supplementary Fig. 13 clear shows

that the spectral shift in the negative ESA signal would not compensate with that of SE, while the GB signal does not change with time.

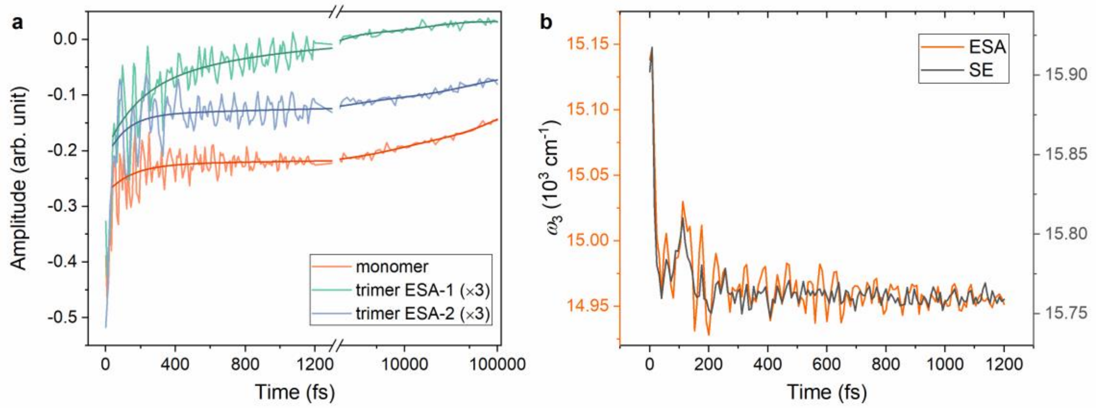

Supplementary Figure 13. Kinetic analysis of ESA signals. (**a**) The kinetics of ESA signal for the monomer (orange) and trimer (green and blue). The three-exponential fitting results are superimposed on top of the original kinetics. The kinetics of trimers were multiplied by a factor of 3 for ease of observation and comparison. (**b**) Peak shift traces of the negative peak (ESA) and the positive peak (SE) at ω_1=16000 cm^{-1}. Both traces show the almost same red-shift process. Source data are provided as a Source Data file.

Supplementary Note 7: Analysis of dynamical Stokes shift data

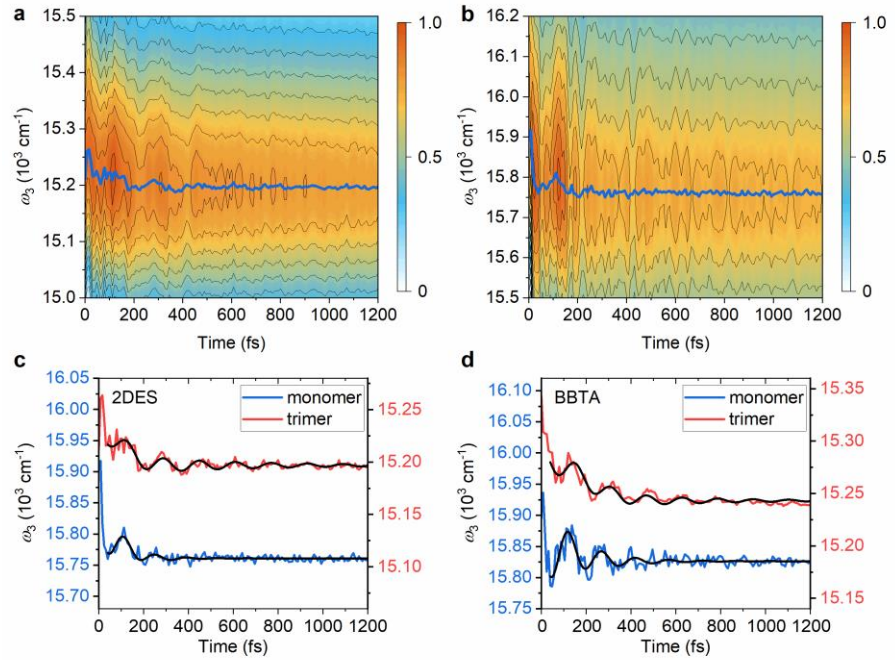

Supplementary Figure 14. Analysis of relaxation kinetics data for the dynamical Stokes shift. The 2D spectra (t_2, ω_3) sliced along the selected excitation frequencies, 15300 cm^{-1} for the trimer (**a**) and 16000 cm^{-1} for the monomer (**b**). The blue lines in (a) and (b) show the time-dependent frequency shift of the maximum peak. The kinetics of the dynamical Stokes shift with fits from 40 fs to 1.2 ps using the 2DES (**c**) and BBTA (**d**) measurements. Source data are provided as a Source Data file.

To extract the dynamical Stokes shift from 2D spectra, we analyzed the time-dependent frequency shift of the maximum peak on the 2D spectra (t_2, ω_3) sliced along the selected excitation frequencies: 15300 cm^{-1} for the trimer and 16000 cm^{-1} for monomer and the two subunits, which correspond to the absorption maxima. The main peak includes the contribution from ground-state bleaching and the stimulated emission, and only the stimulated emission leads to a red shift in the frequency during the spectral evolution. The typical sliced 2D spectra and time-dependent peak frequency changes (blue lines) are shown in Supplementary Figs. 14a and 14b. The frequency resolution of these spectra was improved by spline interpolation of the data. During the first 300 fs, the main peak underwent a significant frequency redshift. Meanwhile, pronounced oscillations were superimposed on the dynamical Stokes shift kinetics. To include the influence of vibrational coherences on dynamical Stokes shift in the lifetime analysis, we combined a Gaussian function and a damped sine function to fit the dynamics[8,9], as shown in Supplementary Fig. 14c. Fitting parameters associated with the vibrational coherences are presented in Supplementary Tables 1 and 2. The dynamical Stokes shift results from BBTA experiments are shown in Supplementary Fig. 14d. In both experiments, the coherence lifetime was longer for the trimer than for the monomer and two subunits, indicating that coherence has a protective effect in the trimer.

Supplementary Table 1. Fitting parameters of the dynamical Stokes shift in the 2DES measurements of the rAPC trimer, monomer, *α*-and *β*-subunits. The fitting function is $f(t)=A_d\exp\left[-t^2/\left(2\tau_d^2\right)\right]+A_c\exp\left(-t/\tau_c\right)\sin\left(2\pi\omega\cdot t+\phi\right)+C$, where τ_d and τ_c are the decay and coherent lifetimes, respectively, A_d and A_c are the corresponding amplitudes, ω (ϕ) is the oscillation frequency (phase), and C is the constant term..

Fitting Parameters	2DES			
	Trimer	Monomer	*α*-subunit	*β*-subunit
A_d (cm^{-1})	32 ± 3	38 ± 6	99 ± 55	55 ± 13
τ_d (fs)	101 ± 7	96 ± 11	45 ± 14	101 ± 19
A_c (cm^{-1})	11 ± 2	39 ± 11	103 ± 41	60 ± 15
τ_c (fs)	**501 ± 104**	**123 ± 30**	**93 ± 19**	**220 ± 59**
ω (cm^{-1})	208 ± 3	242 ± 11	212 ± 12	232 ± 7
ϕ (rad)	2.8 ± 0.2	2.5 ± 0.3	3.1 ± 0.4	2.4 ± 0.3
C (cm^{-1})	15196.9 ± 0.4	15760.7 ± 0.6	15757.1 ± 0.5	15802.5 ± 1.5

Supplementary Table 2. Fitting parameters of the dynamical Stokes shift in the BBTA measurements of the rAPC trimer, monomer, *α* and *β* subunits. The fitting function is same with the 2DES measurements.

**Fitting Parameters**	**BBTA**			
	**Trimer**	**Monomer**	***α*-subunit**	***β*-subunit**
A_d **(cm⁻¹)**	40 ± 2	24 ± 7	48 ± 9	18 ± 5
τ_d **(fs)**	165 ± 7	130 ± 29	87 ± 11	159 ± 39
A_c **(cm⁻¹)**	14 ± 3	63 ± 11	68 ± 12	64 ± 11
τ_c **(fs)**	**401 ± 83**	**170 ± 32**	**176 ± 28**	**162 ± 30**
ω **(cm⁻¹)**	211 ± 3	223 ± 7	247 ± 4	220 ± 7
ϕ **(rad)**	1.7 ± 0.2	2.7 ± 0.3	1.9 ± 0.2	2.4 ± 0.3
C **(cm⁻¹)**	15243.2 ± 0.5	15826.3 ± 1.0	15805.8 ± 0.8	15932.3 ± 1.0

Supplementary Note 8: Supplementary HD-TG and BBTA measurement data

8.1 HD-TG/BBTA spectra and coherent oscillations

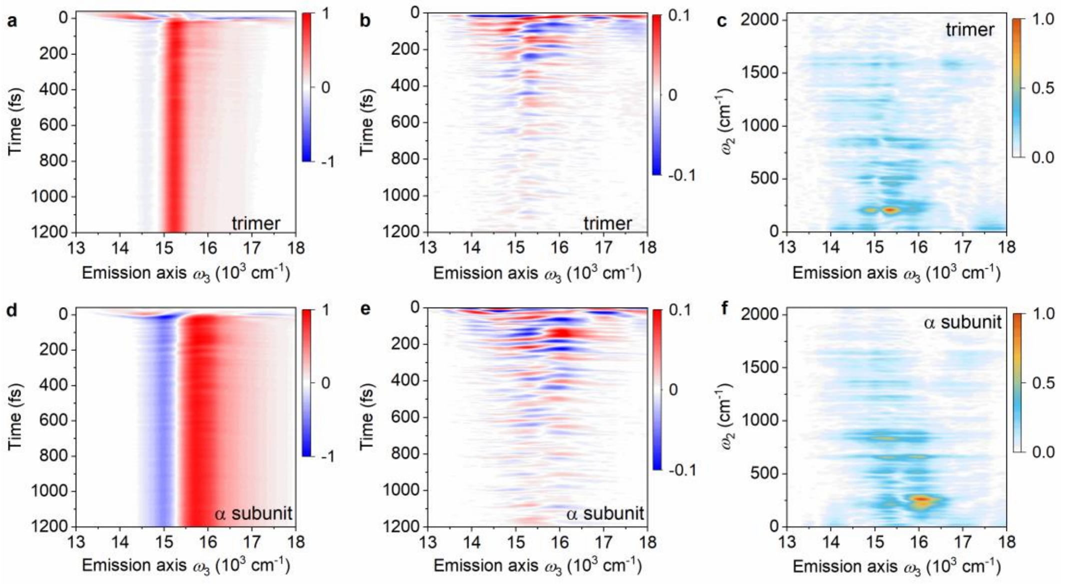

Supplementary Figure 15. HD-TG spectra and coherent oscillations. The phased HD-TG spectra of the rAPC trimer (**a**) and α-subunit (**d**) from -40 fs to 1.2 ps. The GB/SE and ESA signals are indicated in red and blue, respectively. The residual spectra of the trimer (**b**) and α-subunit (**e**) after the subtraction of the population decay using global fitting analysis. The corresponding coherence spectra of (**b**) and (**e**) are shown in (**c**) and (**f**), respectively. Source data are provided as a Source Data file.

In parallel-polarized HD-TG experiments, the time delay between the first two pulses was fixed at zero. The fluence of the excitation pulses was same as that in the 2D measurement. The

HD-TG spectra were phased using the projection slice theorem to match the BBTA results under the same conditions[10]. In parallel-polarized BBTA experiments, the first and third pulses were blocked in the 2D setup. We used the second and fourth pulses as the pump and probe pulses, respectively. To enhance the signal-to-noise ratio of the BBTA measurement, the probe intensity was balanced by a reference beam intensity to eliminate the influence of laser fluctuations during the experiment[11]. The waiting time of HD-TG/BBTA was scanned from -40 fs to 1.2 ps in an 8-fs step. Fourier transformation along t_2 was performed from 40 fs to 1.2 ps to eliminate the effect of overlapping pulse on the coherence analysis. Thus, the actual frequency resolution along ω_2 was ~28 cm^{-1}, while the displayed ω_2 resolution for all FT spectra was set to 7 cm^{-1} after zero-padding.

To filter the sample scattering contribution in BBTA measurements that appears as spectrally resolved fringes on each transient spectra owing to broad bandwidth of the pump pulse, we perform an inverse Fourier transform of the spectra at each time delay, which transforms the frequency domain data into time domain. In the time-domain, a Tukey window was applied over the signal with a fixed bandwidth to selectively filter out the sample scattering. A fast Fourier transform of the scatter-free time-domain signal converts the data back to the frequency domain.

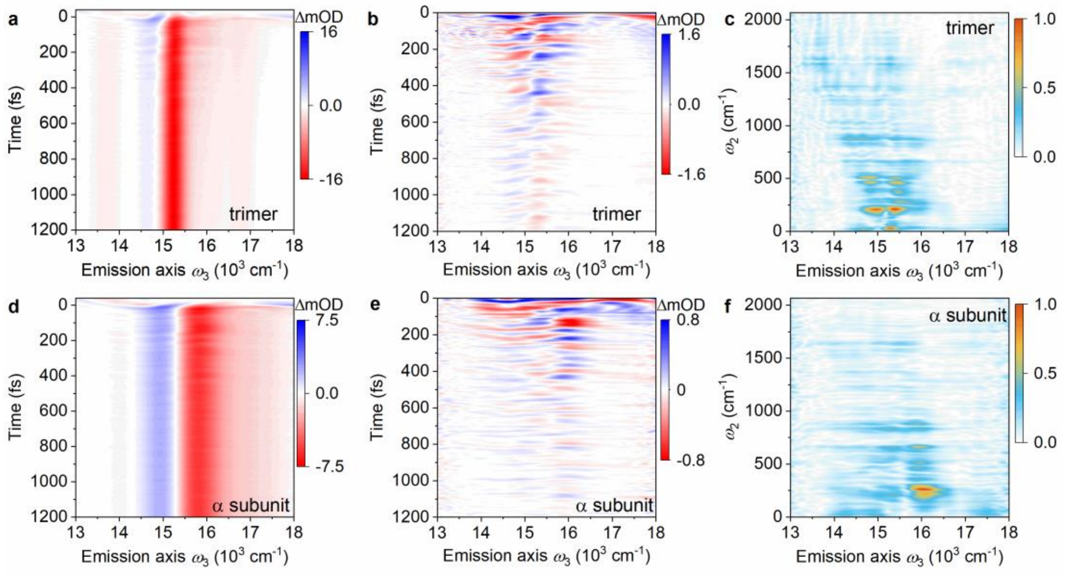

Supplementary Figure 16. BBTA spectra and coherent oscillations. The BBTA spectra of the rAPC trimer (**a**) and α-subunit (**d**) from -40 fs to 1.2 ps. The GB/SE and ESA signals are indicated in red and blue, respectively. The residual spectra of the trimer (**b**) and α-subunit (**e**) after the subtraction of the population decay using global fitting analysis. The corresponding coherence spectra of (**b**) and (**e**) are shown in (**c**) and (**f**), respectively. Source data are provided as a Source Data file.

Supplementary Figures. 15 and 16 display the HD-TG and BBTA spectra revealing the general coherence features of both the rAPC trimer and α subunit. The phased HD-TG spectra (Supplementary Figs. 15a and 15d) provide similar spectral information as the BBTA measurement (Supplementary Fig. 16). The oscillations were mainly superimposed on the GB and

SE signals, while the oscillation contributions in the excited-state absorption region were negligible. The maximal oscillation amplitude reached 10% of the population signal, as shown in Supplementary Figs. 15b and 15e, but it decayed within 500 fs. FT maps of both the rAPC trimer and α-subunit (Supplementary Figs. 15c and 15f) revealed many discrete coherences from 200 to 1600 cm^{-1}. The similar oscillatory frequencies between the trimer and α-subunit suggest that most of the coherences in the trimer are also of vibrational origin because of the exclusive vibrational contributions from the α-subunit observed in the FT maps. The FT amplitude distribution depended on the Huang-Rhys factors of each vibrational mode coupled to the electronic state. In addition, the coherent amplitude of high-frequency vibrations (>1000 cm^{-1}) was easily modulated by adjusting the laser spectral profile.

A comparison of integrated coherence spectra among these three measurements (2DES, HDTG, and BBTA) is shown in Supplementary Fig. 17. All measurements exhibit identical features when the trimer is compared to the monomer and the two subunits. The noise level for each measurement was estimated by taking the average FT amplitude of the four rAPC samples in the high-frequency region from 1800 cm^{-1} to 2100 cm^{-1}, where there is no contribution from coherent vibrational modes. The noise levels for these three measurements are comparable at 15% (2DES), 13% (HD-TG) and 19% (BBTA), respectively. More importantly, quantitative estimation of the coherent amplitude reduction ratio (Table 1 in the main text) confirms the significant suppression of coherent amplitude by approximately 50% at two frequencies, 660 and 805 cm^{-1}, located in the excitonic energy resonant region.

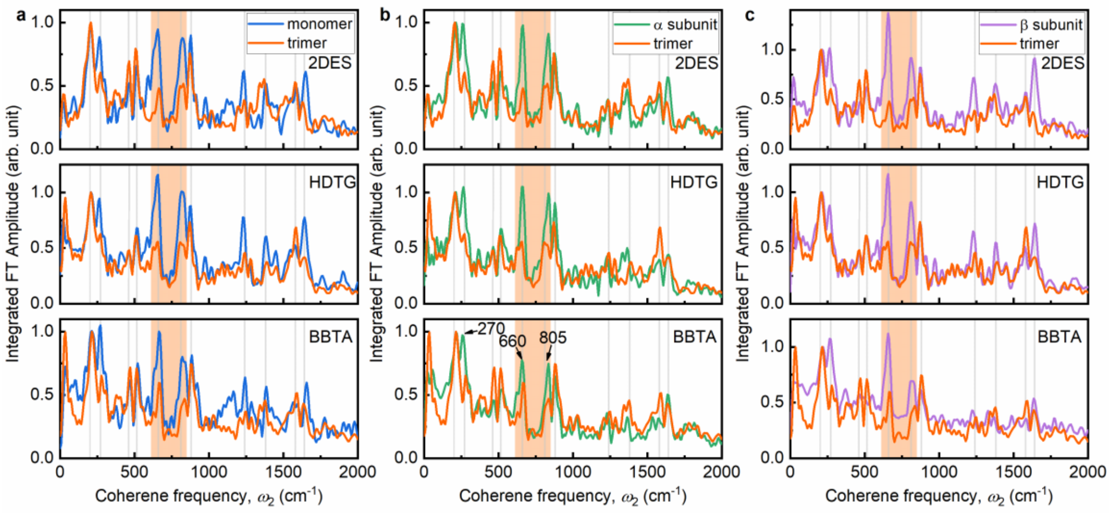

Supplementary Figure 17. Comparison of integrated coherence spectra among three measurements. The three measurements are 2DES (top), HDTG (middle), and BBTA (bottom). The coherence spectra of the rAPC trimer, monomer (**a**), *α*-subunit (**b**), and *β*-subunit (**c**) are shown in orange, blue, green and purple, respectively. Gray lines indicate vibrational modes observed in both spectra: 200, 270, 465, 515, 660, 805, 880, 1240, 1380, 1580, and 1640 cm^{-1}. The orange shaded area shows the near-resonant frequency region in the trimer. All spectra were

normalized by the amplitude at 200 cm^{-1}. Source data are provided as a Source Data file.

8.2 HD-TG measurements with broader laser spectra

To reduce the influence of the laser spectral profile on the coherence amplitudes of high-frequency modes, we used two different laser pulses with broader spectral bandwidth (Supplementary Fig. 18a) to measure the coherence signal of the rAPC samples. We compressed the pulse durations to ~7 fs using a pair of chirped mirrors (Layertec) and a fused silica prism pair. Although high-order dispersions in the form of satellite pulses were more prominent in the TG-FROG measurements in Supplementary Fig. 18 compared with those in Supplementary Fig. 6, the integrated coherence spectra were not affected because these dispersions mainly affect only the early dynamics in the range of pulse width as well as spectral shape. Fourier transformation along t_2 was performed from 40 fs to 1136 fs to eliminate the influence of high-order dispersions of pulses. Thus, the actual frequency resolution along ω_2 was ~30 cm^{-1}, while the displayed ω_2 resolution for all FT spectra was set to ~7.5 cm^{-1} after zero-padding. The integrated coherence spectra of the trimer and α-subunit are shown at the bottom of Fig. 4a in the main text.

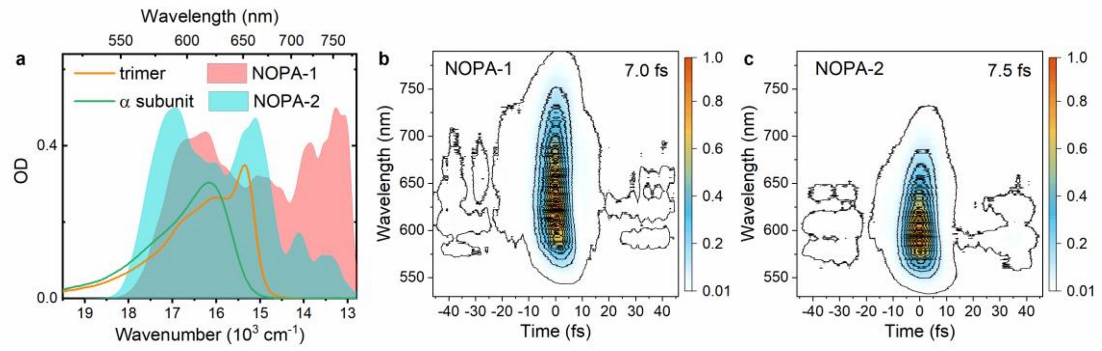

Supplementary Figure 18. HD-TG measurements with a broader laser spectrum. (**a**) Absorption spectra of the rAPC trimer (orange) and α-subunit (green). The laser spectrum of NOPA-1 was used in the HD-TG measurement of the trimer, and the spectrum of NOPA-2 was used in the HD-TG measurement of the α-subunit. The pulse durations of NOPA-1 (**b**) and NOPA-2 (**c**) were characterized by TG-FROG measurements and displayed at the top right of the panels. Contour lines are drawn in 10% intervals. To show the effect of pulse chirp, we set the first level of the contour lines at 0.01. Source data are provided as a Source Data file.

Supplementary Note 9: Supplementary assignments of vibrational modes

Assignments for each vibrational frequency are given in Supplementary Table 3 according to a previous report on phycocyanobilin (PCB) and its analogues[12,13].

Supplementary Table 3. Vibrational frequencies observed in the experiment with the corresponding mode character.

Frequency (cm^{-1})	Mode Character
**465**	Vinyl torsion

**660**	Mixed hydrogen out-of-plane (HOOP) bending, methine torsions, and ring rotations.
**805**	HOOP wag
**1240**	Vinyl, C-H rock
**1380**	C-H and N-H rock<br>C-N and C-C stretching
**1580**	C=NH$^+$ stretching
**1640**	C=C stretching

Supplementary Note 10: Rephasing coherence maps for the rAPC trimer and α-subunit

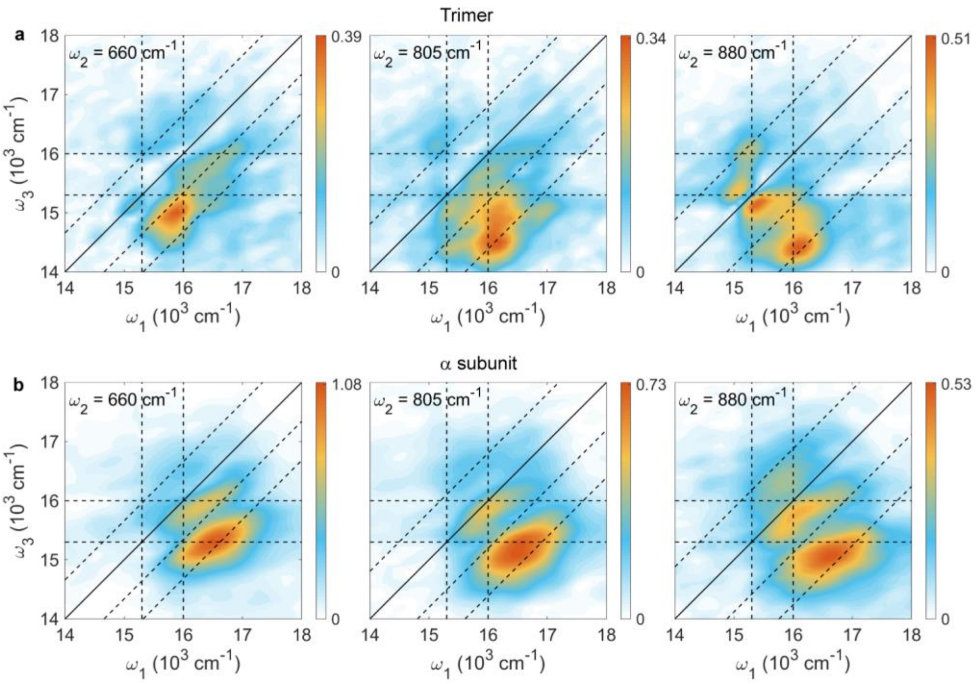

Supplementary Figure 19. Comparison of rephasing coherence maps at resonant frequencies between the rAPC trimer and α-subunit. (**a**) Rephasing coherence maps of the trimer at 660, 805, and 880 cm^{-1}. The vertical/horizontal dashed lines indicate the upper and lower excitonic energies of the trimer. Dashed lines parallel to the diagonal are offset from the diagonal by $\pm\omega_2$ and $-2\omega_2$. (**b**) Rephasing coherence maps of the monomer at 660, 805, and 880 cm^{-1}. Source data are provided as a Source Data file.

Supplementary Figure19 shows the real-part rephasing coherence maps at 660, 805, and 880 cm^{-1} for the trimer and α subunit. The coherence maps for these samples share the same feature, i.e., the intensity of the below-diagonal peak is much larger than that of the above-diagonal peak for all the three frequencies. There is an asymmetrical intensity distribution of the two cross-peaks

located above and below the diagonal in the rephasing coherence map, with a substantial enhancement of the ground-state vibration for both the trimer and α-subunit, where 660 and 805 cm^{-1} are in resonance with the electronic splitting, while 880 cm^{-1} may be in a quasi-resonance state. This clearly indicates that ground-state enhancement in the coherence map cannot be an indicator of the exciton-vibrational coupling.

Supplementary Note 11: Detailed descriptions of the theoretical model

11.1 Dimeric model system in terms of collective vibrational coordinates

Consider a dimer consisting of excited states $\left|e_{i=1,2}\right\rangle$ with energies $\varepsilon_{i=A,B}$, where subscripts A and B denote the two individual molecules in the dimer. The two monomers interact via a dipole-dipole coupling of strength J and are locally coupled to a quantized intramolecular vibration of energy $\omega_{i=A,B}$ with strength $g_{i=A,B} = \hbar\omega_i\sqrt{S}$ where $S = \frac{1}{2}\Delta^2$ is the Huang-Rhys factor and Δ is the displacement of the corresponding nuclear coordinate from its equilibrium state. Assuming the Heitler–London approximation, the Hamiltonian of the dimer is given by[14]

$$\begin{aligned}\hat{H} &= T + \left[V_A(q_A) + V_B(q_B)\right]\left|\mathrm{g}\right\rangle\left\langle g\right| \\ &+ \left[\varepsilon_A + V_A(q_A^*) + V_B(q_B)\right]\left|e_1\right\rangle\left\langle e_1\right| \\ &+ \left[\varepsilon_B + V_A(q_A) + V_B(q_B^*)\right]\left|e_2\right\rangle\left\langle e_2\right| \\ &+ J\left[\left|e_1\right\rangle\left\langle e_2\right| + \left|e_2\right\rangle\left\langle e_1\right|\right]\end{aligned} \tag{1.1}$$

where T is the kinetic energy, $\left|\mathrm{g}\right\rangle$ is the electronic common ground state, q_i is the ground state dimensionless coordinates, and $q_i^* = q_i + \Delta$ is the excited state dimensionless coordinates of the ith molecule. The electronic excitation results in a shift along the dimensionless coordinate q of the potential energy surface defined in the ground state as $V_i(q) = \frac{\hbar\omega_i}{2}q^2$.

The Schrödinger equation for the electronic states at the fixed nuclear configuration is solved as follows:

$$\hat{H}(q_A, q_B)\Psi(q_A, q_B) = E(q_A, q_B)\Psi(q_A, q_B) \tag{1.2}$$

The corresponding secular equation is as follows:

$$\begin{vmatrix} E-\left(\frac{\hbar\omega_A}{2}q_A{}^2+\frac{\hbar\omega_B}{2}q_B{}^2\right) & 0 & 0 \\ 0 & E-\left(\varepsilon_1+\frac{\hbar\omega_A}{2}q_A^{*2}+\frac{\hbar\omega_B}{2}q_B{}^2\right) & J \\ 0 & J & E-\left(\varepsilon_2+\frac{\hbar\omega_A}{2}q_A{}^2+\frac{\hbar\omega_B}{2}q_B^{*2}\right) \end{vmatrix}=0 \tag{1.3}$$

Thus, we get three eigen energies, which are the energy of the common ground state (E_0) and the energies of the two electronic states ($E_\pm$).

$$E_0=\frac{\hbar\omega_A}{2}q_A{}^2+\frac{\hbar\omega_B}{2}q_B{}^2 \tag{1.4}$$

$$\begin{aligned} E_\pm &= E_0+\frac{\varepsilon_A+\varepsilon_B}{2}+\frac{\hbar\Delta}{4}\left[(\omega_A q_A^*+\omega_B q_B^*)+(\omega_A q_A+\omega_B q_B)\right] \\ &\pm\sqrt{\left(\frac{\varepsilon_A-\varepsilon_B}{2}+\frac{\hbar\Delta}{4}\left[(\omega_A q_A^*-\omega_B q_B^*)+(\omega_A q_A-\omega_B q_B)\right]\right)^2+J^2} \end{aligned} \tag{1.5}$$

By assuming that the energy of the common ground state is zero ($E_0 = 0$) and introducing the intermolecular symmetric collective coordinate $Q_s=\frac{1}{\sqrt{2}}(q_A+q_B)$ and the anti-symmetric coordinate $Q_a=\frac{1}{\sqrt{2}}(q_A-q_B)$, if $\omega=\omega_A=\omega_B$, we can simplify the eigen energies of the two electronic states ($E_\pm$) as follows:

$$E_\pm=\frac{\varepsilon_A+\varepsilon_B}{2}+\frac{\hbar\omega\sqrt{S}}{2}\left[Q_s^*+Q_s\right]\pm\sqrt{\left[\frac{\Delta_s}{2}+\frac{\hbar\omega\sqrt{S}}{2}(Q_a^*+Q_a)\right]^2+J^2} \tag{1.6}$$

Under the condition of weak electronic coupling J and a significant site energy difference $\Delta_s=\varepsilon_A-\varepsilon_B$, i.e., $\Delta_s/2>>J$, the eigen energies can be further simplified as follows:

$$E_\pm=\frac{\varepsilon_A+\varepsilon_B}{2}+\frac{\hbar\omega\sqrt{S}}{2}\left[Q_s^*+Q_s\right]\pm\left[\frac{\Delta_s}{2}+\frac{\hbar\omega\sqrt{S}}{2}(Q_a^*+Q_a)\right] \tag{1.7}$$

11.2 Exciton-vibration Hamiltonian of a dimeric model system with Jaynes Cummings form

Jaynes Cummings model can cast the Hamiltonian for wave functions within a local basis into that of a delocalized basis. The total Hamiltonian for wave functions of a local basis without the common ground state is

$$\begin{aligned} \hat{H} &= T+\left[\varepsilon_A+V_A(q_A^*)+V_B(q_B)\right]|e_1\rangle\langle e_1| \\ &+\left[\varepsilon_B+V_A(q_A)+V_B(q_B^*)\right]|e_2\rangle\langle e_2| \\ &+J\left[|e_1\rangle\langle e_2|+|e_2\rangle\langle e_1|\right] \end{aligned} \tag{2.1}$$

For diagonalizing $H_{el}=\begin{pmatrix}\varepsilon_A & J \\ J & \varepsilon_B\end{pmatrix}$, we introduce a rotation operator, which is unitary:

$$U(\theta)=\begin{pmatrix}\cos\theta & \sin\theta\\ -\sin\theta & \cos\theta\end{pmatrix} \tag{2.2}$$

where $\theta=\frac{1}{2}\arctan\left(\frac{2J}{\Delta_s}\right)$ is referred to as the mixing angle and is an effective measure of delocalization of the electronic sub-system or the exciton size, where $\Delta_s=\varepsilon_A-\varepsilon_B$ is the site energy difference.

The diagonalized H_{el} becomes:

$$H'_{el}=E_1|E_1\rangle\langle E_1|+E_2|E_2\rangle\langle E_2| \tag{2.3}$$

where $E_{i=1.2}=\frac{(\varepsilon_A+\varepsilon_B)+(-1)^i\sqrt{\Delta_s{}^2+4J^2}}{2}$ is the eigenvalue of H_{el} and $\begin{pmatrix}|E_1\rangle\\ |E_2\rangle\end{pmatrix}=U^{\dagger}(\theta)\begin{pmatrix}|e_1\rangle\\ |e_2\rangle\end{pmatrix}$ is the delocalized new basis vector after diagonalizing.

Then, the final exciton-vibration Hamiltonian written in the Jaynes-Cummings form under the new basis vector is:

$$\begin{aligned}H_{JC}&=T+\frac{1}{2}\hbar\omega_A q_A^2+\frac{1}{2}\hbar\omega_B q_B^2\\&+\frac{1}{2}\left[(\varepsilon_A+\varepsilon_B)+\frac{\hbar\Delta}{2}(\omega_A q_A^*+\omega_B q_B^*)+\frac{\hbar\Delta}{2}(\omega_A q_A+\omega_B q_B)\right]I\\&+\frac{1}{2}\left[\frac{\hbar\Delta}{2}[(\omega_A q_A^*-\omega_B q_B^*)+(\omega_A q_A-\omega_B q_B)]\cos 2\theta-\sqrt{\Delta_s{}^2+4J^2}\right]\sigma_z\\&+\frac{1}{2}\left[\frac{\hbar\Delta}{2}[(\omega_A q_A^*-\omega_B q_B^*)+(\omega_A q_A-\omega_B q_B)]\right]\sin 2\theta\sigma_x\end{aligned} \tag{2.4}$$

where $I=|E_1\rangle\langle E_1|+|E_2\rangle\langle E_2|=\begin{pmatrix}1&0\\0&1\end{pmatrix}$ is the identity matrix, $\sigma_z=|E_1\rangle\langle E_1|-|E_2\rangle\langle E_2|=\begin{pmatrix}1&0\\0&-1\end{pmatrix}$, $\sigma_x=|E_1\rangle\langle E_2|+|E_2\rangle\langle E_1|=\begin{pmatrix}0&1\\1&0\end{pmatrix}$.

By introducing the intermolecular symmetric collective coordinate $Q_s=\frac{1}{\sqrt{2}}(q_A+q_B)$ and the anti-symmetric coordinate $Q_a=\frac{1}{\sqrt{2}}(q_A-q_B)$, if $\omega=\omega_A=\omega_B$, we can simplify the exciton-vibration Hamiltonian as follows:

$$
\begin{aligned}
H_{JC} &= T+\frac{1}{2}\hbar\omega\left(Q_s^2+Q_a^2\right) \\
&+\frac{1}{2}\left[\left(\varepsilon_A+\varepsilon_B\right)+\hbar\omega\sqrt{S}(Q_s^*+Q_s)\right]I \\
&+\frac{1}{2}\left[\hbar\omega\sqrt{S}(Q_a^*+Q_a)\cos 2\theta-\sqrt{\Delta_s{}^2+4J^2}\right]\sigma_z \\
&+\frac{1}{2}\hbar\omega\sqrt{S}(Q_a^*+Q_a)\sin 2\theta\sigma_x
\end{aligned} \tag{2.5}
$$

where the exciton-vibrational interaction is

$$
H_{\text{coupling}} = \frac{1}{2}\hbar\omega\sqrt{S}\sin\left(2\theta\right)(Q_a^*+Q_a)\sigma_x \tag{2.6}
$$

Eq. (2.6) clearly shows that only the anti-symmetric collective modes can be coupled to the two delocalized excitonic levels.

An approximate indicator describing the degree of coherent excitation transfer is derived from the transition probability between the two exciton-vibration states dominating electronic excitation transfer in the prototype dimer, which is an estimate of the maximum amplitude *A* for the population oscillations expressed as[15]

$$
A=\frac{1}{1+\left(\dfrac{\Delta_s-\omega}{2g\sin 2\theta}\right)^2} \tag{2.7}
$$

where g is the corresponding vibrational strength. The equation shows that the resonant vibrational modes give rise to a maximum amplitude, and any detuning between the electronic splitting and the vibrational energy ($\Delta_s-\omega$) would reduce the oscillation amplitude. On the other hand, a larger amount of electronic delocalization, i.e., a larger $\sin\left(2\theta\right)$, would also lead to a larger oscillation amplitude.

11.3 Dynamical Stokes shift in large polyatomic molecules in the condensed phase derived from a theory for femtosecond pump-probe spectroscopy

Based on the proposed theory for femtosecond pump-probe spectroscopy of large polyatomic molecules in the condensed phase, where a multimode Brownian oscillator model is used to account for high-frequency molecular vibrational and local intermolecular modes as collective solvent motion, Yan and Mukamel provided a semi-classical picture using the density matrix in Liouville space[16,17]. Within this frame, the pump field creates a doorway state that propagates for a specific time interval (waiting time), and the spectrum is calculated by finding its overlap with the window state. In particular, the doorway and window states are wave packets in the phase space.

12.3.1 Correlation function of the *j*th vibration mode within the Brownian oscillator under ultrafast pump-probe conditions

For a molecular model system with electronic-vibration interaction, the Hamiltonian can be expressed as follows:

$$H_g = \tfrac{1}{2}\sum_j \hbar\omega_j \left(p_j^2 + q_j^2\right) \tag{3.1}$$

$$H_e = \tfrac{1}{2}\sum_j \hbar\omega_j \left[p_j^2 + (q_j + d_j)^2\right] + \hbar\omega_{eg} \tag{3.2}$$

where p_j is the dimensionless momentum, q_j is the coordinate of the *j*th nuclear mode with frequency ω_j, and d_j is the dimensionless displacement of the equilibrium configurations of this mode in the two electronic potential surfaces. q_j may represent an intramolecular vibration, an intermolecular liberation, or a collective solvent motion.

Then assume that each mode experiences Brownian motion with a time-dependent Langevin friction function

$$\hat{\gamma}_j(t) = k_B T \left\langle f_j(t) f_j(0) \right\rangle \tag{3.3}$$

where $f_j(t)$ represents the Gaussian stochastic random force due to solvent motion acting on the *j*th mode:

$$\begin{cases} \dot{q}_j = \omega_j p \\ \dot{p}_j = -\omega_j (q_j + \xi d_j) - \int_0^t dt' \hat{\gamma}_j (t - t') p_j(t') + f_j(t) \end{cases} \tag{3.4}$$

where the parameter ξ=0 for the ground-state evolution and ξ=1 for the excited-state evolution.

The correlation function of the *j*th mode within the Brownian oscillator model can be derived as follows:

$$M_j(t) = \frac{\tfrac{1}{2}\left\langle q_j(t) q_j + q_j q_j(t) \right\rangle - \left\langle q_j \right\rangle^2}{\left\langle q_j^2 \right\rangle - \left\langle q_j \right\rangle^2} \tag{3.5}$$

or

$$M(t) = L^{-1}\left[\frac{s + \gamma_j(s)}{s^2 + s\gamma_j(s) + \omega_j^2}\right] \tag{3.6}$$

where L^{-1} denotes the inverse Laplace transform, $\gamma_j(s)$ is the Langevin friction in the Laplace space, and *s* is the Laplace variable conjugate to time *t*:

$$\gamma(s) = \int_0^\infty dt \exp(-st) \hat{\gamma}(t) \tag{3.7}$$

In this model, the time evolution of the *j*th mode depends entirely on its correlation function $M_j(t)$, which in turn depends on its harmonic frequency ω_j and the friction function $\gamma_j(s)$.

It can be found that $M_j(t)$ satisfies the equation of motion:

$$\ddot{M}_j(t) + \int_0^t dt' \hat{\gamma}_j(t-t')\dot{M}_j(t') + \omega_j^2 M_j(t) = 0 \tag{3.8}$$

The frequency dependence of $\gamma_j(s)$ reflects the time scales of the thermal motions of the bath responsible for the random force. If these motions are very fast compared with the oscillator motion, the *s* dependence of $\gamma_j(s)$ is very weak and can be neglected, i.e.,

$$\gamma_j(s) = \gamma_j(s=0) \equiv \gamma_j \tag{3.9}$$

In this case by performing the inverse Laplace transform in Eq. (3.6), we get

$$M_j(t) = e^{-\frac{\gamma_j t}{2}}\left(\cos\overline{\omega}_j t + \frac{\gamma_j}{2\overline{\omega}_j}\sin\overline{\omega}_j t\right), \quad \gamma_j < 2\omega_j \tag{3.10}$$

for an underdamped oscillator, where $\overline{\omega}_j = \left[\omega_j^2 - (\gamma_j/2)^2\right]^{1/2}$.

11.3.2 Comparison of the Langevin equations for $q(t)$ and $M(t)$

When the solvation process is considered, the intra-molecular coordinate of the *j*th nuclear mode q_j can represent local or collective solvent motion as well as intramolecular vibration and intermolecular liberation. In this case, we further assume that each mode experiences Brownian motion with a time-dependent Langevin friction function $\hat{\gamma}_j(t)$. Here, we have

$$\left.\begin{aligned} \dot{q}_j &= \omega_j p \\ \dot{p}_j &= -\omega_j q_j - \int_0^t dt' \hat{\gamma}_j(t-t') p_j(t') + f_j(t) \end{aligned}\right\} \tag{3.11}$$

for the ground state, and

$$\dot{p}_j^* = -\omega_j q_j^* - \int_0^t dt' \hat{\gamma}_j(t-t') p_j^*(t') + f_j(t) \tag{3.12}$$

for the excited state, where p and p^* are the momenta of the intramolecular mode and q and q^* are the intramolecular coordinates for the ground and the excited states, respectively. In addition, we have

$$\left\langle f_j(t) f_k(0) \right\rangle = 2\gamma_j (\overline{p^2})_{jj} \delta(t) \delta_{jk} \tag{3.13}$$

in which $\left(\overline{p^2}\right)_{jj}$ is the thermally averaged momentum square at temperature *T*. Then we get

$$\ddot{q}_j(t) + \int_0^t dt' \hat{\gamma}_j(t-t')\dot{q}_j(t') + \omega_j^2 q_j(t) = f(t) \tag{3.14}$$

for the ground state and

$$\ddot{q}_j^*(t) + \int_0^t dt' \hat{\gamma}_j(t-t')\dot{q}_j^*(t') + \omega_j^2 q^*{}_j(t) = f(t) \tag{3.15}$$

for the excited state. Under the underdamped condition, if we take the approximation that $f(t) \approx 0$, then the Langevin equations for $q(t)$ and $q^*(t)$ have the exact form as that for the

correlation function of $M(t)$; therefore, the solutions for $M(t)$, $q(t)$, and $q^*(t)$ are all the same:

$$q(t) = q^*(t) = Ae^{-\frac{\gamma_j t}{2}}\left(\cos(\bar{\omega}_j t+\varphi_j)+\frac{\gamma_j}{2\bar{\omega}_j}\sin(\bar{\omega}_j t+\varphi_j)\right)$$
$$\approx Ae^{-\frac{\gamma_j t}{2}}\left(\cos(\omega_j t+\varphi_j)+\frac{\gamma_j}{2\omega_j}\sin(\omega_j t+\varphi_j)\right) \tag{3.16}$$

where φ is the initial phase and A is an arbitrary constant. For the current experiment, in the high-frequency region (>500 cm^{-1}), $\gamma << \omega_j$, and we have $\bar{\omega}_j \approx \omega_j$.

11.3.3 Dynamical Stokes shift for the dimeric exciton in the condensed phase

We have shown that the upper and lower energy levels of the dimeric exciton can be expressed as

$$E_+ \approx \frac{\varepsilon_A+\varepsilon_B}{2}+\frac{\Delta_s}{2}+\sqrt{S}\hbar\omega\left[(Q_s^*+Q_a^*)+\left(Q_s+Q_a\right)\right]$$
$$E_- \approx \frac{\varepsilon_A+\varepsilon_B}{2}-\frac{\Delta_s}{2}+\sqrt{S}\hbar\omega\left[(Q_s^*-Q_a^*)+\left(Q_s-Q_a\right)\right] \tag{3.17}$$

In the condensed phase, Q_s (Q_s^*) and Q_a (Q_a^*) experience Brownian motions, and their time-dependent evolution is shown by their respective $Q(t)$ values. Compared with the upper excitonic level, the lower level has a longer lifetime and can be determined experimentally. Thus, the dynamical Stokes shift of the lower exciton level for *j*th mode can be expressed as:

$$\Delta E_-(t) = \frac{\hbar\omega\sqrt{S_j}}{2}\left[\left(Q_{sj}^*(t)-Q^*_{aj}(t)\right)+\left(Q_{sj}(t)-Q_{aj}(t)\right)\right] \tag{3.18}$$

Substituting the underdamped nuclear motion of Eq. (3.16) into Eq. (3.18), finally, we have

$$\Delta E_-^j(t) = A_0^*\sqrt{S_j}\hbar\omega_j e^{-\frac{\gamma_j t}{2}}\sin\left(\frac{\varphi_s^*-\varphi_a^*}{2}\right)\left[-\sin\left(\omega_j t+\frac{\varphi_s^*+\varphi_a^*}{2}\right)+\frac{\gamma_j}{2\omega_j}\cos\left(\omega_j t+\frac{\varphi_s^*+\varphi_a^*}{2}\right)\right]$$
$$+A_0\sqrt{S_j}\hbar\omega_j e^{-\frac{\gamma_j t}{2}}\sin\left(\frac{\varphi_s-\varphi_a}{2}\right)\left[-\sin\left(\omega_j t+\frac{\varphi_s+\varphi_a}{2}\right)+\frac{\gamma_j}{2\omega_j}\cos\left(\omega_j t+\frac{\varphi_s+\varphi_a}{2}\right)\right] \tag{3.19}$$

where the superscript "*" refers to the excited-state properties. Eq 8 shows that the amount of dynamical Stokes shift depends on the phase relationship between the two intermolecular collective modes of the excited and ground states. In particular, when the motion of both collective coordinates Q_s (Q_s^*) and Q_a (Q_a^*) are correlated (i.e., $\varphi_s-\varphi_a=0$ or $\varphi_s^*-\varphi_a^*=0$), the coherent contribution of the *j*th mode to dynamical Stokes shift will be thoroughly suppressed at the lower excitonic state.

Supplementary References

1 Liu, S. *et al.* Biosynthesis of fluorescent cyanobacterial allophycocyanin trimer in *Escherichia coli*. *Photosynth. Res.* **105**, 135-142 (2010).

2 Li, W. *et al.* Efficient purification protocol for bioengineering allophycocyanin trimer with N-terminus Histag. *Saudi J. Biol. Sci.* **24**, 451-458 (2017).

3 Csatorday, K., MacColl, R., Csizmadia, V., Grabowski, J. & Bagyinka, C. Exciton interaction in allophycocyanin. *Biochemistry* **23**, 6466-6470 (1984).

4 Beck, W. F. & Sauer, K. Energy-transfer and exciton-state relaxation processes in allophycocyanin. *J. Phys. Chem.* **96**, 4658-4666 (1992).

5 Moya, R., Norris, A. C., Kondo, T. & Schlau-Cohen, G. S. Observation of robust energy transfer in the photosynthetic protein allophycocyanin using single-molecule pump-probe spectroscopy. *Nat. Chem.* (2022).

6 Edington, M. D., Riter, R. E. & Beck, W. F. Evidence for coherent energy-transfer in allophycocyanin trimers. *J. Phys. Chem.* **99**, 15699-15704 (1995).

7 Zhang, J. M. *et al.* Investigations of Ultrafast Exciton Dynamics in Allophycocyanin Trimer. *J. Phys. Chem. A* **105**, 8878-8891 (2001).

8 Jimenez, R., Fleming, G. R., Kumar, P. V. & Maroncelli, M. Femtosecond solvation dynamics of water. *Nature* **369**, 471-473 (1994).

9 Jumper, C. C. *et al.* Broad-band pump-probe spectroscopy quantifies ultrafast solvation dynamics of proteins and molecules. *J. Phys. Chem. Lett.* **7**, 4722-4731 (2016).

10 Jonas, D. M. Two-dimensional femtosecond spectroscopy. *Annu. Rev. Phys. Chem.* **54**, 425-463 (2003).

11 Feng, Y., Vinogradov, I. & Ge, N. H. General noise suppression scheme with reference detection in heterodyne nonlinear spectroscopy. *Opt. Express* **25**, 26262-26279 (2017).

12 Mroginski, M. A., Mark, F., Thiel, W. & Hildebrandt, P. Quantum mechanics/molecular mechanics calculation of the Raman spectra of the phycocyanobilin chromophore in α-C-phycocyanin. *Biophys. J.* **93**, 1885-1894 (2007).

13 Andel, F. *et al.* Probing the photoreaction mechanism of phytochrome through analysis of resonance Raman vibrational spectra of recombinant analogues. *Biochemistry* **39**, 2667-2676 (2000).

14 Butkus, V., Zigmantas, D., Abramavicius, D. & Valkunas, L. Distinctive character of electronic and vibrational coherences in disordered molecular aggregates. *Chem. Phys. Lett.* **587**, 93-98 (2013).

15 Siwiak-Jaszek, S. & Olaya-Castro, A. Transient synchronisation and quantum coherence in a bio-inspired vibronic dimer. *Faraday Discuss.* **216**, 38-56 (2019).

16 Yan, Y. J. & Mukamel, S. Electronic dephasing, vibrational-relaxation, and solvent friction in molecular nonlinear optical-line shapes. *J. Chem. Phys.* **89**, 5160-5176 (1988).

17 Yan, Y. J. & Mukamel, S. Femtosecond pump-probe spectroscopy of polyatomic molecules in condensed phases. *Phys. Rev. A* **41**, 6485-6504 (1990).
